# Supplementary material for: Sharks associated with a large sand shoal complex: Community insights from longline and acoustic telemetry surveys
Source: PLoS One. 2023 Jun 16;18(6):e0286664. doi: 10.1371/journal.pone.0286664 (PMC10275426; doi:10.1371/journal.pone.0286664)
Supplement: S1 Table — Values are mean with range in parentheses. The mean and range across all longline sets is also provided. (DOCX) [file pone.0286664.s001.docx]

**S1 Table. Habitat conditions under which each shark species was collected on longline sets**. Values are mean with range in parentheses. The mean and range across all 978 longline sets is also provided.

| **Species** | **Water Depth (m)** | **Seafloor Slope (m)** | **Distance To Shore (km)** | **Water Temp (°C)** | **Salinity (psu)** | **Dissolved Oxygen (mg/l)** | **Water Clarity (Secchi, m)** |
| --- | --- | --- | --- | --- | --- | --- | --- |
| Sharpnose Shark | 11.9 (2.7-18.6) | 1.2 (0.0-5.8) | 7.6 (0.3-15.6) | 25.6 (14.1-30.7) | 36.1 (33.1-39.2) | 6.8 (5.1-9.5) | 6.4 (0.4-17.0) |
| Blacknose Shark | 8.3 (2.5-18.6) | 1.4 (0.0-6.7) | 3.3 (0.1-14.8) | 23.4 (16.0-30.5) | 35.7 (33.5-38.7) | 6.9 (4.1-9.2) | 2.8 (0.8-15.0) |
| Blacktip Shark | 7.4 (1.8-17.7) | 1.2 (0.0-6.4) | 3.2 (0.1-12.3) | 22.1 (16.4-29.1) | 35.7 (33.8-38.1) | 7.2 (5.2-9.2) | 2.3 (0.8-15.0) |
| Finetooth Shark | 6.2 (1.8-17.7) | 1.5 (0.0-5.6) | 1.9 (0.1-11.6) | 21.2 (16.6-29.5) | 36.0 (33.8-38.3) | 7.1 (4.5-8.2) | 1.8 (0.7-5.3) |
| Nurse Shark | 8.7 (2.1-17.8) | 1.2 (0.0-5.8) | 6.0 (0.2-15.6) | 25.2 (20.4-28.9) | 36.1 (34.6-38.7) | 7.0 (5.7-8.3) | 5.2 (0.9-17.0) |
| Bonnethead Shark | 8.7 (2.8-16.5) | 1.5 (0.0-5.2) | 3.7 (0.4-11.9) | 20.6 (15.8-28.4) | 35.2 (34.0-37.4) | 7.4 (5.4-9.2) | 2.4 (0.9-4.0) |
| Spinner Shark | 9.0 (3.5-17.7) | 1.7 (0.0-7.0) | 4.7 (0.4-12.0) | 26.0 (19.0-29.8) | 36.0 (34.4-37.7) | 6.5 (5.4-7.9) | 2.4 (1.0-6.5) |
| Scalloped Hammerhead | 13.5 (5.9-18.6) | 0.9 (0.0-4.1) | 7.5 (0.6-14.8) | 23.7 (20.4-28.6) | 36.3 (34.1-38.7) | 6.9 (5.9-7.5) | 4.2 (0.8-8.5) |
| Lemon Shark | 5.4 (2.4-13.7) | 1.1 (0.0-4.4) | 1.2 (0.2-11.6) | 18.1 (16.4-22.3) | 35.1 (34.0-38.6) | 7.7 (6.5-8.3) | 2.0 (0.9-4.3) |
| Sandbar Shark | 11.0 (4.6-16.2) | 1.8 (0.5-6.7) | 5.9 (0.4-14.8) | 18.4 (16.2-23.8) | 35.0 (34.1-36.6) | 8.2 (6.8-9.2) | 2.7 (1.1-4.6) |
| Great Hammerhead | 9.1 (4.3-17.2) | 0.7 (0.0-1.7) | 6.8 (0.5-12.3) | 24.6 (19.3-28.7) | 36.3 (34.3-38.7) | 7.1 (6.5-8.8) | 4.3 (1.5-8.9) |
| Bull Shark | 7.8 (5.5-11.9) | 2.0 (0.3-4.9) | 2.2 (0.4-9.5) | 20.2 (16.6-25.6) | 34.7 (33.8-35.8) | 7.1 (6.7-7.4) | 4.4 (1.7-15.0) |
| Sand Tiger Shark | 9.1 (4.9-14.9) | 0.9 (0.6-1.3) | 3.6 (0.4-11.2) | 19.2 (17.1-26.4) | 35.0 (34.5-35.8) | 7.4 (6.1-8.1) | 2.8 (1.6-3.9) |
| Tiger Shark | 13.1 (4.7-17.0) | 0.9 (0.3-1.8) | 8.2 (3.4-12.9) | 23.5 (20.8-27.6) | 36.2 (35.5-37.2) | 7.2 (6.7-7.9) | 5.5 (0.8-11.1) |
| Smooth Hammerhead | 8.9 (8.9-8.9) | 1.9 (1.9-1.9) | 7.1 (7.1-7.1) | 17.2 (17.2-17.2) | 33.7 (33.7-33.7) | 8.5 (8.5-8.5) | 2.0 (2.0-2.0) |
| Dusky Shark | 10.3 (10.3-10.3) | 2.5 (2.5-2.5) | 7.6 (7.6-7.6) | 21.0 (21.0-21.0) | 36.3 (36.3-36.3) | 7.4 (7.4-7.4) | 7.1 (7.1-7.1) |
| Unknown Carcharhinid | 10.1 (3.2-13.6) | 2.3 (0.4-5.2) | 6.9 (0.2-13.2) | 19.0 (15.8-22.6) | 36.1 (35.2-37.0) | 7.8 (7.6-8.0) | 3.0 (2.0-4.3) |
| **All Longline Sets** | **8.7 (1.5-20.6)** | **1.3 (0.0-8.2)** | **4.9 (0.1-15.7)** | **23.4 (14.1-3.07)** | **36.0 (33.1-39.2)** | **7.0 (4.1-9.6)** | **3.3 (0.4-17.0)** |
