# Supplementary material for: Sharks associated with a large sand shoal complex: Community insights from longline and acoustic telemetry surveys
Source: PLoS One. 2023 Jun 16;18(6):e0286664. doi: 10.1371/journal.pone.0286664 (PMC10275426; doi:10.1371/journal.pone.0286664)
Supplement: S2 Table — Detections from offshore reef stations are excluded. Values are mean with range in parentheses. (DOCX) [file pone.0286664.s002.docx]

**S2 Table. Habitat conditions under which acoustically tagged sharks were detected on the Canaveral Shoals**. Detections from offshore reef stations are excluded. Values are mean with range in parentheses.

| **Species** | **Water Depth (m)** | **Seafloor Slope (m)** | **Distance From Shore (km)** | **Water Temp (°C)** | **Water Clarity (K490^1^)** | **Sediment % Fines** | **Sediment % Organics** |
| --- | --- | --- | --- | --- | --- | --- | --- |
| Blacknose Shark | 7.4 (1.4-16.0) | 4.8 (0.9-13.4) | 1.8 (0.2-13.7) | 24.5 (16.4-30.1) | 0.24 (0.1-1.9) | 6.0 (0.0-15.8) | 1.3 (0.3-2.5) |
| Blacktip Shark | 9.5 (1.4-16.0) | 5.3 (0.9-13.4) | 4.8 (0.2-13.7) | 22.9 (17.1-29.6) | 0.25 (0.1-1.9) | 3.3 (0.0-15.8) | 1.1 (0.3-2.5) |
| Bonnethead Shark | 9.9 (1.4-16.0) | 4.6 (0.9-13.4) | 5.3 (0.2-13.7) | 20.1 (17.2-29.4) | 0.36 (0.2-1.2) | 3.8 (0.0-15.8) | 1.2 (0.3-2.5) |
| Bull Shark | 9.3 (1.4-16.0) | 4.7 (0.9-13.4) | 5.1 (0.2-13.7) | 23.9 (16.4-29.5) | 0.20 (0.1-1.0) | 1.6 (0.0-15.8) | 1.0 (0.3-2.5) |
| Common Thresher Shark | 10.1 (8.1-12.1) | 3.5 (3.0-4.0) | 6.8 (3.1-10.5) | 20.6 (20.6-20.7) | - | 0.1 (0.1-0.2) | 1.2 (0.8-1.5) |
| Finetooth Shark | 7.0 (1.4-16.0) | 5.2 (0.9-13.4) | 2.4 (0.2-13.7) | 22.2 (17.1-29.9) | 0.24 (0.1-1.8) | 2.6 (0.0-15.8) | 0.9 (0.3-2.5) |
| Great Hammerhead | 8.0 (1.4-16.0) | 4.6 (1.2-13.4) | 4.2 (0.2-13.7) | 27.2 (22.9-29.5) | 0.07 (0.1-0.1) | 1.5 (0.0-12.6) | 1.0 (0.3-1.9) |
| Lemon Shark | 6.7 (1.4-16.0) | 7.1 (0.9-13.4) | 1.5 (0.2-13.7) | 22.4 (16.4-29.9) | 0.24 (0.1-1.2) | 1.5 (0.0-15.8) | 0.8 (0.3-2.5) |
| Nurse Shark | 9.7 (3.7-16.0) | 5.8 (1.5-13.4) | 7.1 (0.2-13.7) | 27.5 (20.2-29.6) | 0.10 (0.1-0.2) | 0.9 (0.0-8.4) | 1.0 (0.3-1.7) |
| Sand Tiger | 8.7 (1.4-16.0) | 5.8 (0.9-13.4) | 3.6 (0.2-13.7) | 19.5 (16.5-24.3) | 0.27 (0.2-0.5) | 3.7 (0.0-15.8) | 1.1 (0.3-2.5) |
| Sandbar Shark | 11.6 (3.0-16.0) | 3.6 (1.2-11.0) | 7.4 (0.2-13.7) | 21.0 (16.0-24.0) | 0.31 (0.2-0.4) | 2 (0.0-12.6) | 1.2 (0.5-1.9) |
| Scalloped Hammerhead | 8.8 (1.4-16.0) | 6.6 (0.9-13.4) | 3.2 (0.2-13.7) | 26.3 (17.7-29.7) | 0.17 (0.1-0.6) | 4.8 (0.0-15.8) | 1.2 (0.3-2.5) |
| Sharpnose Shark | 10.3 (1.4-16.0) | 3.8 (0.9-13.4) | 6.5 (0.2-13.7) | 26.8 (17.3-30.1) | 0.17 (0.1-1.0) | 1.5 (0.0-15.8) | 1.1 (0.3-2.5) |
| Spinner Shark | 10.3 (4.7-16.0) | 5.2 (0.9-13.4) | 8.2 (0.2-13.7) | 22.6 (20.3-23.7) | 0.23 (0.2-0.3) | 2.2 (0.0-15.8) | 1.1 (0.4-2.5) |
| Tiger Shark | 10.5 (1.4-16.0) | 4.6 (0.9-13.4) | 7.1 (0.2-13.7) | 26.6 (17.5-30.1) | 0.14 (0.1-0.4) | 2.1 (0.0-15.8) | 1.1 (0.3-2.5) |
| White Shark | 10.6 (3.0-16.0) | 4.3 (0.9-13.4) | 7.8 (0.2-13.7) | 20.6 (16.8-25.1) | 0.23 (0.1-0.6) | 1.1 (0.0-15.8) | 1.0 (0.3-2.5) |
| **All Detections** | **7.8 (1.4-16.0** | **5.1 (0.9-13.4)** | **2.7 (0.2-13.7)** | **23.9 (16.0-30.1)** | **0.2 (0.1-2.9)** | **4.2 (0.0-15.8)** | **1.1 (0.3-2.5)** |
| ^1^Higher values indicate lower water clarity | |  |  |  |  |  |  |
