# Supplementary material for: Sharks associated with a large sand shoal complex: Community insights from longline and acoustic telemetry surveys
Source: PLoS One. 2023 Jun 16;18(6):e0286664. doi: 10.1371/journal.pone.0286664 (PMC10275426; doi:10.1371/journal.pone.0286664)
Supplement: S3 Table — Variance values estimate the amount of variation in the dataset explained by the factor while SD gives the square root of these values, and thus is in Bray–Curtis units. Pairwise tests of community similarity across seasons are also provided. (DOCX) [file pone.0286664.s003.docx]

**Table S3.** PERMANOVA model that tests for shark community differences in longline catch across seasons and depth zones. Variance values estimate the amount of variation in the dataset explained by the factor while SD gives the square root of these values, and thus is in Bray–Curtis units. Pairwise tests of community similarity across seasons are also provided.

| **Source** | | **df** | **SS** | **MS** | **Pseudo F** | ***p*** | **Component** | **Variance** | **SD** |
| --- | --- | --- | --- | --- | --- | --- | --- | --- | --- |
| Season | | 3 | 18975 | 6325 | 8.7 | **0.0001** | Fixed | 534.6 | 23.1 |
| Depth Zone | | 1 | 5523 | 5523 | 7.6 | **0.0002** | Fixed | 229.9 | 15.2 |
| Season X Depth Zone | | 3 | 1764 | 588 | 0.8 | 0.6815 | Fixed | 26.0 | 5.1 |
| Residual | | 34 | 24633 | 725 |  |  |  | 724.5 | 26.9 |
| Total | | 41 | 51052 |  |  |  |  |  |  |
|  |  | |  |  |  |  |  |  |  |
| **Pairwise Tests** | **Similarity** | | | **t** | ***p*** |  |  |  |  |
| Fall vs. Spring | 60.8 | | | 1.8 | **0.0108** |  |  |  |  |
| Fall vs. Summer | 58.8 | | | 2.4 | **0.0002** |  |  |  |  |
| Spring vs. Summer | 56.3 | | | 2.9 | **0.0002** |  |  |  |  |
| Spring vs. Winter | 48.0 | | | 2.5 | **0.0006** |  |  |  |  |
| Fall vs. Winter | 44.2 | | | 3.1 | **0.0001** |  |  |  |  |
| Summer vs. Winter | 37.2 | | | 4.6 | **0.0001** |  |  |  |  |
