# Supplementary material for: Sharks associated with a large sand shoal complex: Community insights from longline and acoustic telemetry surveys
Source: PLoS One. 2023 Jun 16;18(6):e0286664. doi: 10.1371/journal.pone.0286664 (PMC10275426; doi:10.1371/journal.pone.0286664)
Supplement: S5 Table — Groups include the offshore dredge site, offshore control site, all other offshore shoal stations (>1.5 km from shore), offshore reef stations, and nearshore shoal stations (< 1.5 km from shore). (DOCX) [file pone.0286664.s005.docx]

**Table S5.** PERMANOVA model that tests for shark community differences in Canaveral Array detections between the sand dredge site, control site, other nearshore shoal stations (< 1.5 km from shore), other offshore shoal stations (>1.5 km from shore), and reef stations.

| **Source** | **df** | | **SS** | | **MS** | Pseudo F | ***p*** | **Component** | | **Variance** | **SD** |
| --- | --- | --- | --- | --- | --- | --- | --- | --- | --- | --- | --- |
| Habitat Type | 4 | | 4435 | | 1109 | 7.8 | **0.0001** | Fixed | | 110.5 | 10.5 |
| Residual | 43 | | 6092 | | 142 |  |  |  | | 141.7 | 11.9 |
| Total | 47 | | 10527 | |  |  |  |  | |  |  |
|  |  | |  | |  |  |  |  | |  |  |
| **Pairwise Tests** | | **Similarity** | | **t** | ***p*** | **Pairwise Tests** | | | **Similarity** | **t** | ***p*** |
| Control vs. Reef | | 86.0 | | 1.5 | **0.033** | Dredge vs. Reef | | | 80.1 | 2.4 | **0.0152** |
| Control vs. Offshore | | 83.7 | | 1.3 | 0.107 | Dredge vs. Nearshore | | | 79.2 | 2.7 | **0.0001** |
| Reef vs. Offshore | | 82.6 | | 1.5 | **0.021** | Nearshore vs. Offshore | | | 77.9 | 3.4 | **0.0001** |
| Dredge vs. Offshore | | 81.6 | | 1.5 | **0.0443** | Reef vs. Nearshore | | | 74.3 | 3.8 | **0.0001** |
| Dredge vs. Control | | 80.7 | | 2.8 | **0.0068** | Control vs. Nearshore | | | 74.1 | 4.2 | **0.0001** |
