# Supplementary material for: Sharks associated with a large sand shoal complex: Community insights from longline and acoustic telemetry surveys
Source: PLoS One. 2023 Jun 16;18(6):e0286664. doi: 10.1371/journal.pone.0286664 (PMC10275426; doi:10.1371/journal.pone.0286664)
Supplement: Attachment — Submitted filename: PONE-Feb2023.pdf [file pone.0286664.s007.pdf]

Review Comments to the Author
Please use the space provided to explain your answers to the questions above. You may also include additional comments for the author, including concerns about dual publication, research ethics, or publication ethics. (Please upload your review as an attachment if it exceeds 20,000 characters) (Limit 200 to 20000 Characters)

1. Is the manuscript technically sound, and do the data support the conclusions?
The manuscript must describe a technically sound piece of scientific research with data that supports the conclusions. Experiments must have been conducted rigorously, with appropriate controls, replication, and sample sizes. The conclusions must be drawn appropriately based on the data presented.

2. Has the statistical analysis been performed appropriately and rigorously?

3. Have the authors made all data underlying the findings in their manuscript fully available?
The PLOS Data policy requires authors to make all data underlying the findings described in their manuscript fully available without restriction, with rare exception (please refer to the Data Availability Statement in the manuscript PDF file). The data should be provided as part of the manuscript or its supporting information, or deposited to a public repository. For example, in addition to summary statistics, the data points behind means, medians and variance measures should be available. If there are restrictions on publicly sharing data—e.g. participant privacy or use of data from a third party—those must be specified.

4. Is the manuscript presented in an intelligible fashion and written in standard English?
PLOS ONE does not copyedit accepted manuscripts, so the language in submitted articles must be clear, correct, and unambiguous. Any typographical or grammatical errors should be corrected at revision, so please note any specific errors here.

Other comments:

This manuscript makes use of long-term longline catch data and passive acoustic telemetry to describe the shark assemblages of a sand bank system and investigate the temporal and spatial patterns in their distribution and abundance. The manuscript also aims to identify environmental factors that affect shark assemblages on sand banks, and to compare shark assemblages between undisturbed systems and sand banks where dredging is taking place. The manuscript therefore sets out to accomplish a lot, though the long-term datasets available and the robust statistical treatment of the data on the author's part allow for it.

The data is robustly collected, and the study set up allows the authors to make good inference about the seasonal and spatial trends of shark diversity and abundance at the study site. The combination of fishery-dependent and -independent methods strengthen the conclusions further. The statistical analysis is well planned and executed, lending credibility to the results. Sometimes the explanation of analytical techniques and decisions isn't sufficiently clear/explicit, but can easily be made more accessible in review (see line-by-line comments below).
The results are mostly well presented, but the scope and variety of objectives sometimes results in parts being rushed and not sufficiently explained (see comments below). In particular, not enough attention is given to the comparison between the dredged and undisturbed site, despite this being one of the main aims of the manuscript. The role of the site as a nursery is also a key section in the discussion, but is not properly covered in the results. A better match between key points in the discussion and results sections would make this manuscript stronger.
The discussion is mostly well presented but, as suggested above, somewhat suffers from trying to achieve too much. The manuscript would probably benefit from a slightly more cohesive vision for the discussion. Some sections are not discussed in enough detail or aren't supported by the results enough, while others could be avoided (see detailed comments below). The discussion would also perhaps benefit from a stronger conclusion. Nonetheless, the structure and language of the manuscript is clear and well thought-out, making for an interesting read.
Altogether, this is a well written manuscript and well executed study. The results presented are interesting and mostly well presented, supported by robust statistical analysis. Minor refinement are needed to hone-in its focus, but otherwise the manuscript is of very good quality.

Line by line comments:
45 – References here could contain the more recent Dulvy lab papers
48 – Instead of "most sharks", specify if you mean shark species or populations
60 – this sentence needs a little more explanation of what the authors mean by "a unique perspective on shark community dynamics", and perhaps some supporting references.
60 – 63 This line of thought is a little rushed and could use further explanation
67 – missing references (after " when compared to adjacent deeper water")
87 – While the introduction flows well, at this point I find myself wondering why these questions are of "central importance". Perhaps a little more detail on the ecological relevance of the aims of the paper and their role in explaining impacts from dredging would help in making this section stronger.
90 – 94 The comparison of methodologies feels more appropriate for the discussion
135 – Could be good to give a summary statistic for soak times (mean and standard deviation)
191 – how were mortality events identified?
202 – How was "shark community" quantified/measured?
203 – It is quite difficult to understand what averaging means in this context – what quantity exactly was averaged?
205 – Would be good to make clearer that study year was not considered a factor ONLY in acoustic data analysis. Sometimes explanation of analytical details for the two methods are hard to distinguish and separate throughout the methods section.

240 – Again, what do the authors mean by averaged here? More detail is necessary
249 – Perhaps a brief explanation of sediment percent organics and percent fine estimates is needed here for audiences who are not familiar with these quantities.
267 – The authors should make it clear that the difference between deep and shallow areas is not apparent when sharpnose shark catches are excluded, and that there is much inter-specific variation in patterns of abundance between depths.
275 – Missing reference to Figure 3
278 – Could the female-skewed sex ratios for cool season sharks have something to do with the existence of nurseries? Perhaps female sharks coming in to pup at this time? This perhaps warrants further attention in results to have stronger ties to the discussion.
The section is also missing a better description of length measurement distributions for the different species, which again would provide a better link to the section on nurseries in the discussion.
281 – The CPUE comparisons are not clear, and the lowercase codes are not explained anywhere so they are impossible to understand. This figure caption needs much more detail to make the figure legible.
293 – 298 This feels a bit more fit for the discussion rather than the results section
344 – change reference to fig 6 with "(Fig. 6 A and B, respectively)"
352 – I think section would benefit from a deeper analysis of the results. For example, the shark communities at dredged and control sites look separate in figure 7, but this isn't touched on in the section. It would also be good to look into residency times at the two sites and report any potential differences. If dredging activities took place during the course of the study, another interesting metric would also be the time between dredging taking place and the first detection of sharks at the site. As the effects (or lack thereof) of dredging sandbanks on shark communities is one of the main foci of the paper, this section really needs a bit more attention.
411 – perhaps, given the very narrow depth range, you could refer to "habitat differences" (ridge vs adjacent waters) rather than "shallow vs deep"?
451 – Change "due to " with "thanks to"
504 – As mentioned above, this section seems, at the moment, a little disconnected from the aims of the paper and the results presented. As it is of interest to the ecology of this habitat, I would suggest that the authors make more explicit reference to it in the introduction and better highlight figures/numbers that pertain to this in the results. For example, the numbers in lines 514-517 are only found in the table but never specifically highlighted in the text. This would also be a good place to reflect on possible links to the highly female-skewed sex ratios registered in cold seasons.
517 – change "underestimating" with "underestimated".
521 – can you give any info/detail on the sizes or estimated ages of these sharks?
526 – The conclusion to this section would be stronger if it focused on ecological/conservation importance of the findings, rather than methodological considerations.
527 – This section feels important, but contains conjectures and/or claims that aren't totally supported by the results at the moment. The data would however allow for a deeper analysis of differences (composition, residency, seasonality) that really is needed to support this section (see some comments above).
557 – it is not really the shark species itself that is designated as an "Habitat Area of Particular concern". Perhaps it would be best to rephrase the sentence slightly to something

like “one of only three shark species in the US to benefit from these designations”. Also, Specify you are referring to BEACH nourishment.
563 – This section feels a little rushed and perhaps doesn’t add as much to the manuscript as other sections do/could. I would suggest that the authors consider removing this section in favour of giving more attention to the sections on nurseries and effects of dredging. This would hone the focus of the manuscript on ecology and conservation and make the work stronger.
587 – The manuscript presents many interesting results on ecology and conservation of sharks in this habitat and geographic area. It is therefore a shame to conclude on technicality of methodologies that are perhaps more obvious. I suggest the authors sacrifice the section above in favour of a stronger conclusion that brings together the results of the study and perhaps offers recommendation on the management of sandbanks for shark conservation.
Fig 1 – Perhaps the dots in A could be changed to make apparent which sets belong to each year of the study?
In B, I suggest the authors make it clearer that the circle of dots further north is the control site.
Fig 2. As mentioned above, I cannot quite understand what the letters above the boxplots are trying to tell me. Also, did you consider varying the width of the boxes by the sample size, or using violin/rainclouds plots to better represent the underlying distribution of the data? The simplicity of boxplots sometimes can mask subtle differences that become apparent when using these other visualisation tools.
Fig 3. I really like the level of detail and ease of comparison offered by the figure. Would it be possible to also add a figure (though I realise there are many already) showing maps of species distributions in the area?
Fig 6. This is a minor comment, but in all other figure you have used these shades of blue to indicate different depths, so it took me a while to realise the colours in this figure actually indicated different sampling strategies. Perhaps the colours could be changed to make the distinction more immediate? The same also applies to figure 8.
Fig 7. In caption, the authors use “borrow” instead of “dredging”. I suggest keeping to the latter for consistency with the rest of the manuscript.
